# Trends and biases in the social cost of carbon

**DOI:** 10.1111/nyas.15340

**Published:** 2025-05-07

**Authors:** Richard S. J. Tol

**Affiliations:** ^1^ Department of Economics University of Sussex Falmer UK; ^2^ Institute for Environmental Studies Vrije Universiteit Amsterdam The Netherlands; ^3^ Department of Spatial Economics Vrije Universiteit Amsterdam The Netherlands; ^4^ Tinbergen Institute Amsterdam The Netherlands; ^5^ CESifo Munich Germany; ^6^ Payne Institute for Public Policy Colorado School of Mines Golden Colorado USA

**Keywords:** citation bias, meta‐analysis, publication bias, social cost of carbon

## Abstract

An updated and extended meta‐analysis confirms that the central estimate of the social cost of carbon is around $200–250/tC ($700–900/tCO_2_) with a large, right‐skewed uncertainty and trending up as ethical views have changed. The pure rate of time preference and the inverse of the elasticity of intertemporal substitution are key assumptions, the total impact of 2.5℃ warming less so. The social cost of carbon is much higher if climate change is assumed to affect economic growth rather than the levels of output and welfare. The literature is dominated by a relatively small network of authors, based in a few countries. Publication and citation bias may have pushed the social cost of carbon up.

## INTRODUCTION

The social cost of carbon is a central statistic in climate policy. It measures the benefit of slightly reducing carbon dioxide emissions, justifying (or not) policies to do that.^1^, ^2^ There is, therefore, a large literature on the social cost of carbon, estimating its size, critiquing estimates, and commenting on its (lack of) application.^3^, ^4^ This paper contributes by updating a previous meta‐analysis^5^ and exploring fields recently added to the meta‐database, coding the assumptions underlying estimates of the social cost of carbon. Furthermore, I study citation and co‐author networks and their influence on published estimates, showing signs of both publication and citation biases.

Previous meta‐analyses focused on the characterization of the uncertainty about the social cost of carbon,^6^, ^7^ the detection of trends,^5^, ^8^ and the discovery of publication bias.^9^, ^10^ I briefly revisit uncertainty and trends as nothing much has changed: The uncertainty about the social cost of carbon is right‐skewed and large, with a thick, perhaps fat fail.^11^ Estimates of the social cost of carbon have increased over time. The analysis below reaffirms these findings using a larger, more refined database.

I shed new light on publication bias. Havránek et al.^9^ use tests for publication bias that are appropriate for regression results—estimates of the social cost of carbon are not. Instead, I follow Tol,^10^ testing for confirmation bias—do later estimates agree or disagree with earlier ones? I improve on that paper in two ways. The newly documented citation network allows me to distinguish between earlier estimates that are surely known to the researchers and those that may not have been. A richer set of assumptions underlying the estimates of the social cost of carbon allows me to test whether researchers were influenced by the *results* of previous studies or by their *assumptions*.

Furthermore, this paper tests for citation bias in the social cost of carbon literature. Citation bias has been studied in other literatures,^12^, ^13^, ^14^ but not for the social cost of carbon. As with publication bias, tests for citation bias focus on significance and are, therefore, not applicable here. Instead, I test on effect size and underlying assumptions.

The paper proceeds as follows. The “Data and Descriptive Statistics” section discusses the descriptive statistics of the extended database, setting up the hypothesis to be tested in the “Analysis” section. The “Conclusion” section discusses the results and their implications.

## DATA AND DESCRIPTIVE STATISTICS

The data have been collected over two decades, starting with the meta‐analysis of Tol.^6^ No restrictions were placed on the type of publication, discipline, or language. The current database extends the one used by Tol^5^ with more papers and more estimates. The updated data also contain more information about the assumed intertemporal welfare functions and the impacts of climate change.^a^ Furthermore, I added fields on the authors and their affiliations, as well as on citations. The database covers the period 1980–2023.

The variable of interest is the social cost of carbon for emissions in 2010, expressed in 2010 US dollars per metric ton of carbon. Data are quality‐weighted: Central estimates are emphasized, robustness checks discounted. Peer‐reviewed papers are weighted more, papers that compute marginals incorrectly^b^ or use synthetic scenarios less. Censoring is conceptual rather than statistical: Estimates that exceed the ability to pay ($7609/tC) are disregarded. Estimates that exceed the Leviathan tax^20^ are partially disregarded using a linear function that equals 1 at $1141/tC and 0 at $7609/tC. Uncensored estimates range from –$1133/tC to $105,886,350/tC. The data are described in Tol.^21^ The database is on GitHub (https://github.com/rtol/metascc/tree/master).

**FIGURE 1:**
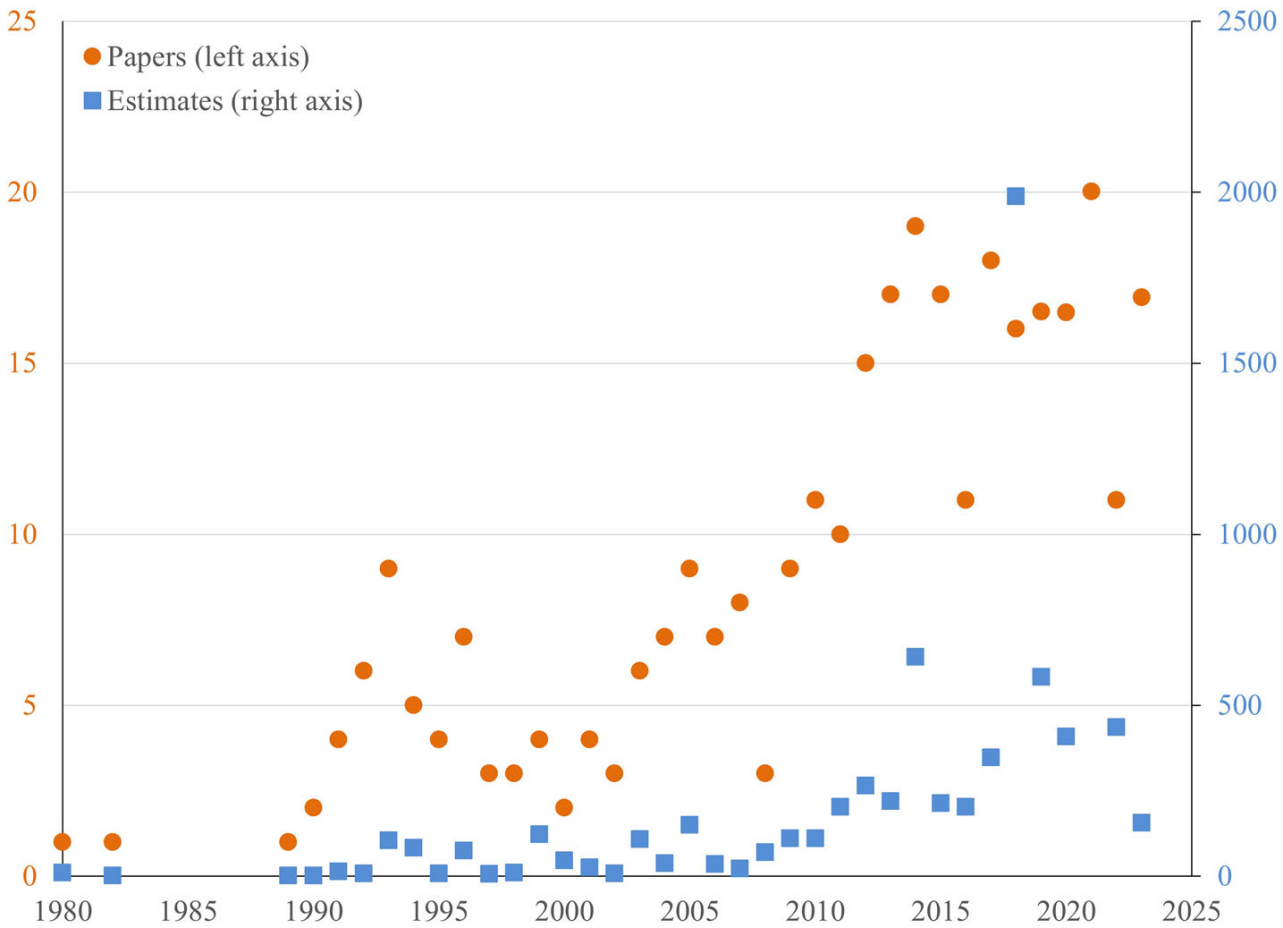
The number of papers on and estimates of the social cost of carbon by year.

Figure 1 shows a steady rise in the number of papers and estimates per year. Three papers were published in the 1980s, 20 in 2021. Those 20 papers contained 5458 estimates of the social carbon. As the literature has grown richer and computers faster, researchers report a wider range of sensitivity analyses.

**FIGURE 2:**
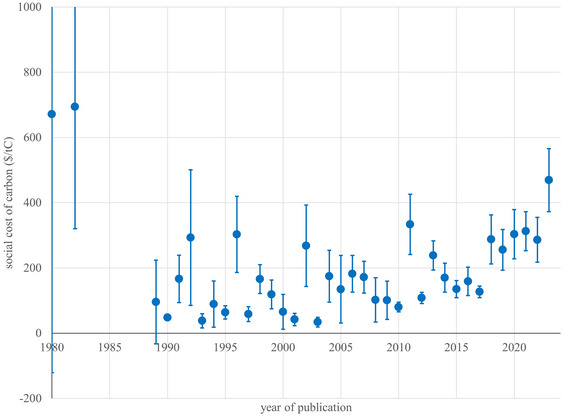
The average social cost of carbon by year of publication. The interval shown is the mean plus and minus the standard deviation. Estimates are author‐ and quality‐weighted and censored.

**FIGURE 3:**
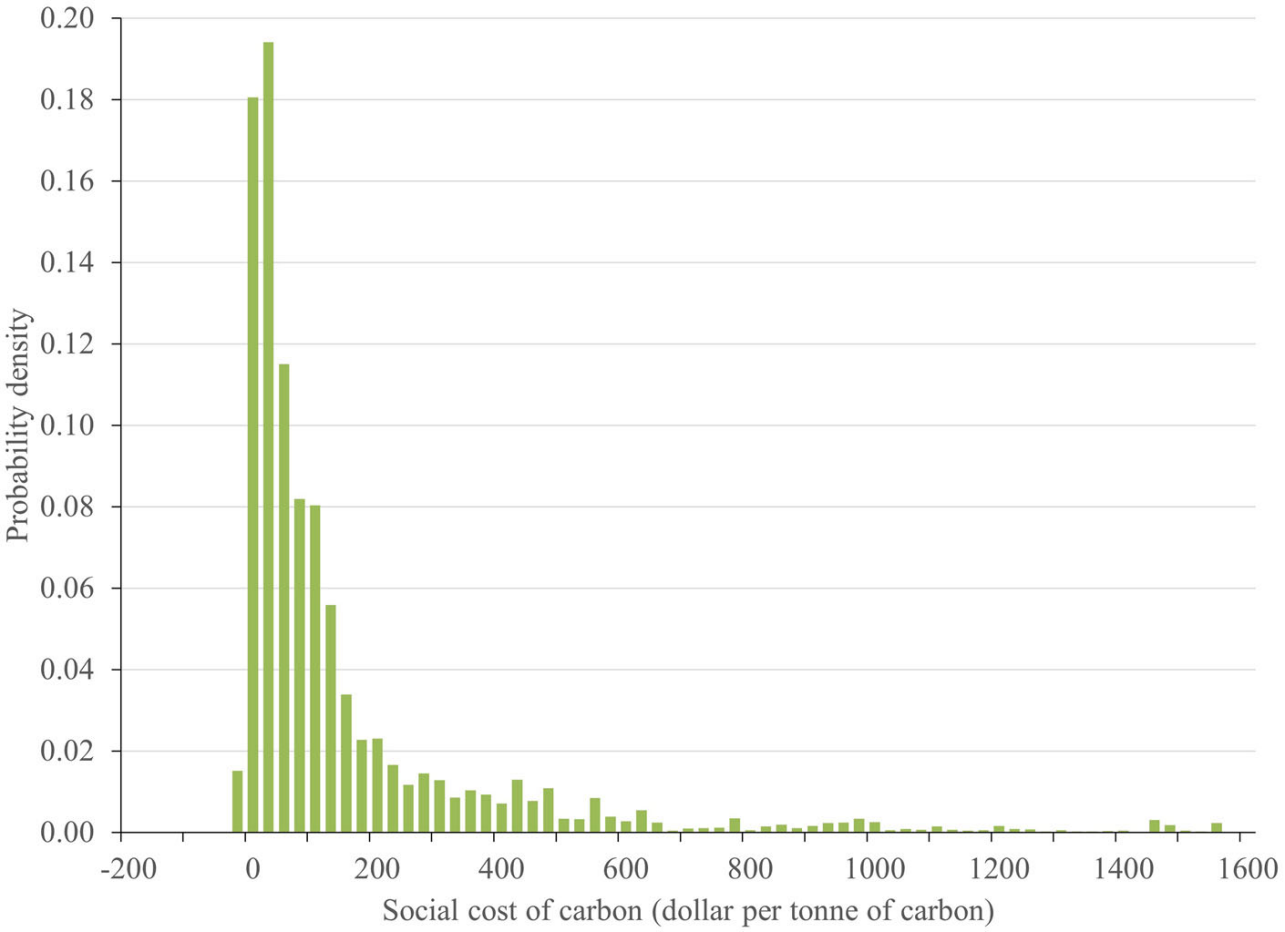
The histogram of published estimates of the social cost of carbon. Estimates are author‐ and quality‐weighted and censored.

Estimates of the social cost of carbon started high, fell, and then rose again after 2010; see Figure 2 and Tol.^5^ The mode of all published estimates lies between $25/tC and $50/tC. The weighted mean is $207/tC; if older studies are discounted at 5% (10%) per year, this increases to $231/tC ($253/tC). The distribution has a pronounced right tail. Only 1.6% of estimates point to a social *benefit* of carbon (Figure 3).

**FIGURE 4:**
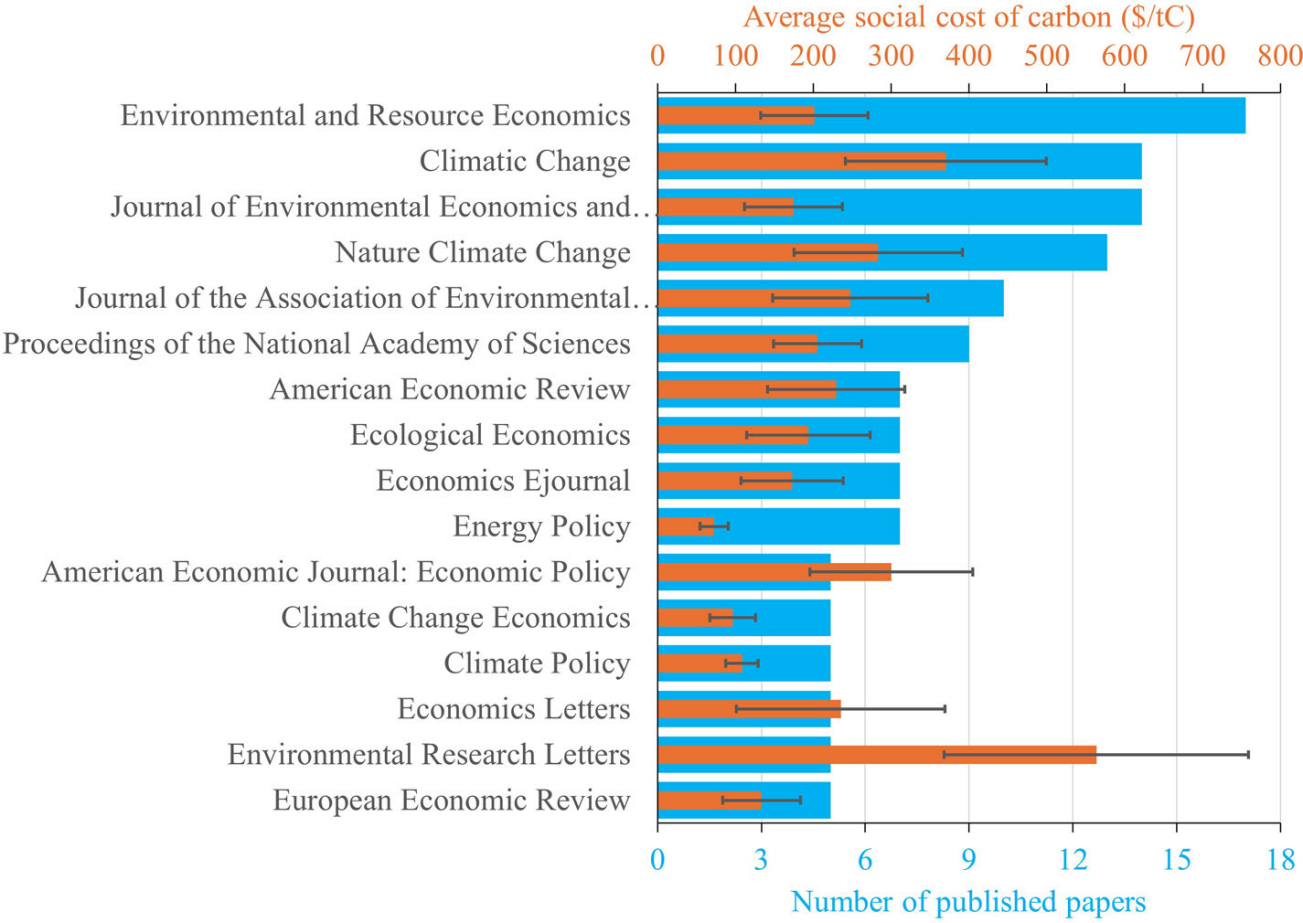
The average social cost of carbon for the 16 journals that published five or more papers.

A new feature of the meta‐database, Figure 4 shows the number of papers published per journal, for the 16 journals that published five or more papers. Figure 4 also shows the average social cost of carbon for these papers. *Environmental & Resource Economics* is the most prolific journal with 17 papers. Estimates of the social cost of carbon are highest, $564/tC, in *Environmental Research Letters*, and lowest in *Energy Policy*, $72/tC. Economics journals tend to publish lower estimates than natural science and environmental policy journals. The difference is on average $86/tC, with a standard error of $44/tC; p=0.027.

Another new feature of the meta‐database: the average paper has 1.03 authors. Estimating the social cost of carbon is a solo activity: Most papers take an existing model and scenarios and tweak a few assumptions. That said, the maximum is 23 authors,^22^ reflecting the effort needed to build a new integrated assessment model and develop new scenarios.

**FIGURE 5:**
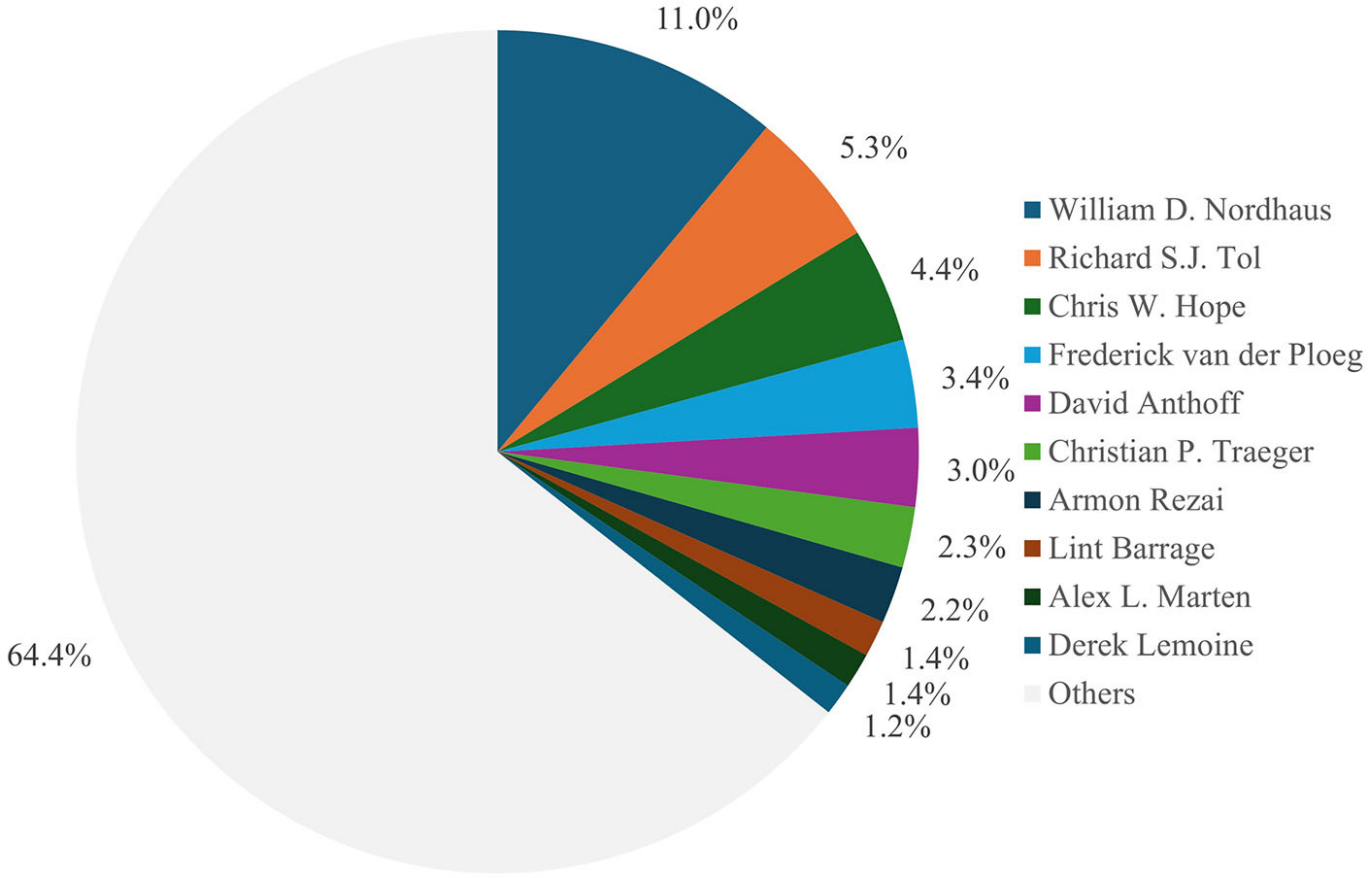
The number of papers on the social cost of carbon by author.

Figure 5 shows the 10 most‐prolific authors, who together cover almost half of the literature. William D. Nordhaus has made the largest contribution, spanning more than four decades.^23^, ^24^

**FIGURE 6:**
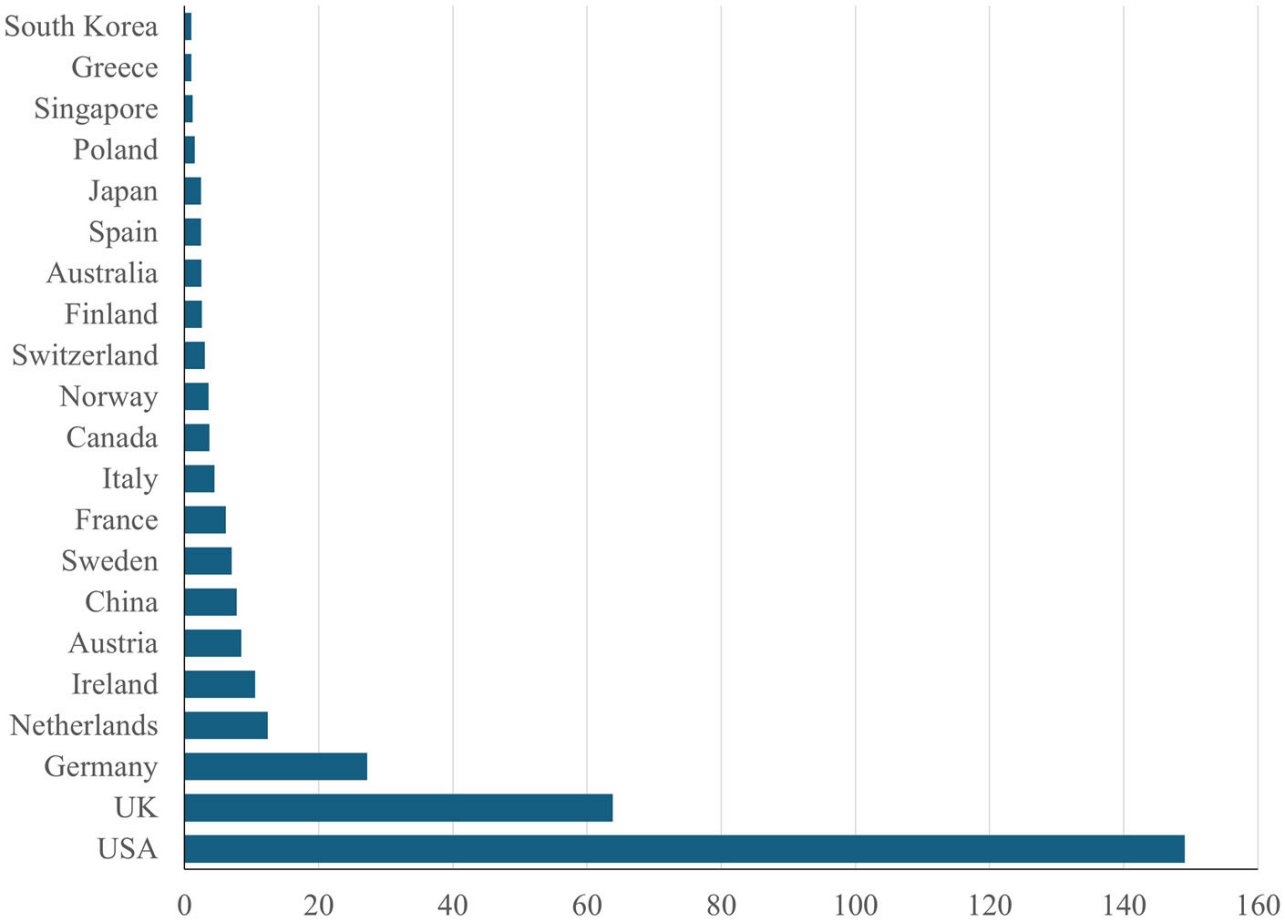
The number of papers on the social cost of carbon by country of affiliation.

Figure 6 shows the country of affiliation of these authors. Almost half of all papers originate in the United States, about a fifth in the UK. Nineteen other countries contributed to this literature. There are few papers from Asia and no papers from Latin America or Africa. This reduces the political legitimacy of estimates of the social cost of carbon. Dong et al.^25^ show that people from the represented countries tend to be more patient and so prefer a higher social cost of carbon.

Figure S1 shows the co‐author networks of authors with five or more papers. There are seven such networks, connecting 160 out of 332 authors. The largest network has 116 members, including the top 5 in Figure 5. There are significant differences between networks. The smallest one, Peck and Teisberg, reports an average social cost of carbon of $32/tC, significantly smaller than any of the other networks. The results for the Budolfson and Newbold networks are statistically indistinguishable. The largest network, Nordhaus', is in the middle with $156/tC. The Traeger network finds a higher social cost of carbon on average, but the difference is statistically insignificant. The two remaining networks, Gerlagh and Groom, are again indistinguishable. Indeed, the variation in Groom's network is so large that its average is not significantly different from that of five of the six other networks. Because of these inconclusive results (and because the Nordhaus and Gerlagh networks will merge in the near future), co‐author *networks* will not be further considered. I instead focus on individual authors below.

**FIGURE 7:**
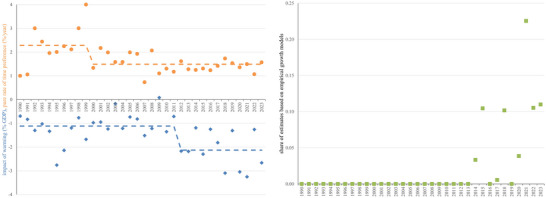
Assumptions for the social cost of carbon by year of publication. Left panel: The orange circles show the assumed pure rate of time preference, the blue diamonds the welfare impact of 2.5℃ warming. In both cases, a regime swift fits the data better than a linear trend. The dashed lines show the before and after means. Right panel: The green squares show the fraction of studies that assume that climate change affects the growth rate of the economy rather than the level of economic output. All results are author‐ and quality‐weighted and censored.

Figure S2 shows the citation network. The node size is weighted arithmetic incloseness, counting not just the citations of a paper, but also citations of citations, citations of citations of citations, and so on. Weights are inversely proportional to the number of citations in the referring paper. The citation network is dense. There are cycles as people cite early versions. I regressed citation number on publication year and identified the 10% of papers that have the largest residuals. These papers are most influential for their age. They are named in Figure S2. The central‐most papers, uncorrected for age, are Nordhaus^26^ and Nordhaus.^27^ Corrected for age, Stern et al.^28^ comes first, followed by Nordhaus.^29^

## ANALYSIS

### Social cost of carbon

The social cost of carbon is typically estimated using a numerical integrated assessment model; the first to do so were Peck and Teisberg.^30^ Golosov et al.^31^ use a starkly simplified model to derive a closed‐form equation for the social cost of carbon.^c^ Bijgaart et al.^33^ built on this for a less unrealistic representation. They show that the assumed impact of climate change and the pure rate of time preference are the key determinants of the social cost of carbon. van den Bremer and van der Ploeg^18^ add a correction factor for risk. These insights guide the analysis below.

Table 1 shows regression results for the social cost of carbon. The base specification includes the pure rate of time preference and the inverse of the elasticity of intertemporal substitution. Higher values for these two parameters imply a higher discount rate^34^ and so a lower social cost of carbon. The estimated coefficients have the correct sign. Time preference is always highly significant, utility curvature is significant in the base and most alternative specifications. In the base specification, a 1 percentage point increase in the pure rate of time preference reduces the social cost of carbon by $112/tC. An increase in the inverse of the elasticity of intertemporal substitution reduces the social cost of carbon by $45/tC. These are large changes for modest changes in assumptions.

**TABLE 1:** Study characteristics and the social cost of carbon.

	SCC	SCC	1/SCC	SCC	SCC	SCC	SCC
PRTP	$-112.3^{***}$	$-265.0^{***}$	−0.014	$-123.0^{***}$	$-75.77^{***}$	$-121.4^{***}$	$-108.2^{***}$
	(13.41)	(36.17)	(0.028)	(14.37)	(17.47)	(13.36)	(13.42)
PRTP^2^		41.90 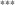					
		(9.31)					
EIS	$-45.10^{**}$	$-59.08^{**}$	0.228 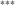	$-71.65^{***}$	$-100.3^{***}$	$-48.38^{***}$	$-54.34^{***}$
	(17.57)	(24.37)	(0.037)	(28.90)	(17.61)	(17.55)	(24.39)
EIS^2^		1.278 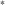					
		(0.912)					
Year	2.513	3.892 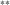	$-0.008^{**}$	0.196	7.506 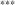	1.849	6.973 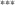
	(1.581)	(1.587)	(0.003)	(1.758)	(2.194)	(1.728)	(2.065)
Impact				$-19.97^{***}$			
				(4.891)			
Linear				$-207.8^{**}$			
				(98.56)			
Quadratic				$-65.00^{*}$			
				(34.25)			
Parabola				$-110.6^{*}$			
				(60.88)			
Weitzman				$-180.9^{**}$			
				(83.76)			
Fund				$-101.3^{**}$			
				(45.36)			
Growth				265.4 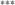			
				(98.03)			
Tax					178.0		
					(162.3)		
$T_{2100}$					−6.923		
					(40.83)		
$\mathrm{Tax}\times T_{2100}$					−53.43		
					(49.21)		
Hope						−10.46	
						(56.52)	
Nordhaus						−58.43	
						(43.88)	
van der Ploeg						$-176.1^{***}$	
						(49.08)	
Tol						$-196.2^{***}$	
						(41.14)	
Traeger						$-131.7^{**}$	
						(56.53)	
Cited SCC							0.140
							(0.105)
Not cited							0.930 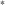
							(0.516)
Aware							0.359 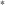
							(0.207)
Constant	−4,635	$-7,302^{**}$	15.1 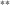	103.0	$-14,699^{***}$	−3,227	$-13,875^{***}$
	(3185)	(3195)	(6.7)	(3538)	(4403)	(3483)	(4213)
Observations	787	787	786	706	393	787	781
*R* ^2^	0.097	0.124	0.052	0.163	0.139	0.136	0.109

*Note*: Standard errors are shown in parentheses; 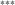 *p* <0.01; 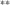 *p* <0.05; 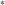 *p* <0.1.

The base specification assumes a linear relationship between these parameters and the social cost of carbon. In a single model, the relationship between pure time preference and the social cost of carbon is closer to *inversely* proportional,[for example, Ref. 33]. However, as some estimates assume a zero pure rate of time preference, I add a quadratic term in column 2 of Table 1. The relationship between the social cost of carbon and the elasticity of the marginal utility of consumption is more complicated,^35^ but I added a quadratic term as a robustness check. In the quadratic specification, the social cost of carbon responds more (less) strongly for small (large) values of the pure rate of time preference and the inverse of the elasticity of intertemporal substitution.

The *inverse* of the social cost of carbon is the dependent variable in the third column of Table 1. The pure rate of time preference is no longer significant. The elasticity of the marginal utility of consumption is significant and appropriately switches sign.

The base specification also includes the year of publication. Estimates of the social cost of carbon have increased over time but the significance of the upward trend varies with specification. In the base specification, changes in the assumed discount rate explain the observed upward trend (Figure 2). The assumed pure rate of time preference shows a downward trend in Figure 7 but a regime shift better explains the data: Before 2000, the average utility discount rate was 2.3%; after that, 1.5%.

Table 1 also shows a number of extended specifications. The fourth column includes the assumed economic impact of 2.5℃ warming. The social cost of carbon increases with the assumed severity of climate change. If the impact of 2.5℃ warming is 1% of GDP worse, the social cost of carbon increases by $20/tC. This too partly explains the upward trend. Figure 7 shows that before 2012, studies on average assumed that 2.5℃ warming would reduce human welfare by the equivalent of losing 1.1% of income; after that, this was revised to 2.1%.

The two breaks in Figure 7, in 2000 and 2012, do not coincide with an identifiable event, such as the publication of DICE (1992), IPCC AR2 (1996), the Stern Review (2006), or the first federal social cost of carbon (2010).

The shape of the impact function matters too. Commonly used impact functions find a lower social cost of carbon than a smorgasbord of “other” or undefined impact functions (the base category)—but the popular impact functions do not lead to results that differ from other often‐used functions.

Studies that adopt the findings of econometric estimates of the impact of weather shocks on economic growth^36^, ^37^ report higher social cost of carbon estimates,^38^, ^39^ on average $265/tC higher. This partly explains the upward trend. Prior to 2014, no estimates of the social cost of carbon were based on these econometric studies.^d^ In 2014, 3% were, rising to 11% in 2023 (Figure 7).

There is no statistically significant impact of imposing the estimated social cost of carbon as a carbon tax (Table 1, column 3). The assumed temperature in 2100 does not affect the reported estimate either, nor does the interaction between temperature and the dummy whether a carbon tax is imposed. (Recall that a carbon tax reduces emissions and so global warming and the social cost of carbon.) A large number of studies do not report results for global warming; the sample size shrinks accordingly. This affects the size but not the sign or significance of the welfare parameters. The time trend becomes significant.

The social cost of carbon is lower in papers (co‐)authored by Rick van der Ploeg, Richard Tol, or Christian Traeger (Table 1, column 4). The differences between these authors are not statistically significant.

Figure 4 shows systematic differences between journals, but these averages do not account for differences in composition. Table 2 regresses the social cost of carbon on the pure rate of time preference, the inverse of the elasticity of intertemporal substitution, the year of publication, and dummies for the 16 journals that published five papers or more on the social cost of carbon. Two journals stand out: *Environmental Research Letters* and *Climatic Change* publish estimates of the social cost of carbon that are statistically significantly higher than do other journals.

**TABLE 2:** Differences between journals.

	SCC	PRTP	EIS	impact	Cited SCC
PRTP	$-115.1^{***}$				
	(13.56)				
EIS	$-45.15^{**}$				
	(17.71)				
Year	2.382	$-0.0236^{***}$	0.00111	$-0.0470^{***}$	$-6.587^{***}$
	(1.699)	(0.00447)	(0.00316)	(0.0112)	(0.534)
*European Econ Rev*	−75.44	0.153	$-0.393^{**}$	0.318	13.56
	(86.65)	(0.234)	(0.176)	(0.894)	(33.49)
*Env Research Let*	333.5 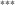	−0.195	−0.115	1.063	4.184
	(93.02)	(0.252)	(0.190)	(0.789)	(34.77)
*Econ Let*	58.51	−0.159	0.481 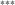	$-1.552^{**}$	−46.22
	(85.42)	(0.231)	(0.174)	(0.649)	(33.10)
*Climate Pol*	−52.72	0.0968	−0.285	0.453	−41.80
	(94.83)	(0.257)	(0.188)	(0.777)	(32.18)
*Climate Change Econ*	−95.43	−0.268	0.0996	1.076	−1.143
	(114.6)	(0.311)	(0.234)	(0.730)	(33.16)
*AEJ Econ Pol*	51.44	0.213	0.329	−1.152	29.58
	(99.59)	(0.270)	(0.203)	(0.712)	(36.24)
*Energy Pol*	74.71	0.860 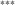	−0.286	−0.735	66.58**
	(116.5)	(0.275)	(0.236)	(0.571)	(28.11)
*Econ Ejournal*	−126.2	$-0.663^{***}$	−0.156	0.508	−35.90
	(87.81)	(0.237)	(0.178)	(0.538)	(27.40)
*Ecol Econ*	69.02	0.607 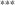	−0.0952	0.738	41.30
	(71.36)	(0.192)	(0.145)	(0.540)	(27.48)
*American Econ Rev*	67.16	0.299	0.217	−0.202	106.3 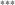
	(87.77)	(0.238)	(0.170)	(0.636)	(32.24)
*Proc Nat Acad Science*	44.02	0.0733	0.373 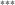	−0.0170	21.95
	(67.64)	(0.183)	(0.132)	(0.589)	(24.98)
*J Assoc Env Res Econ*	27.22	−0.116	0.0312	0.965 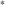	67.61 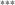
	(79.48)	(0.216)	(0.147)	(0.538)	(24.78)
*Nature Climate Change*	−33.18	0.159	0.255 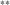	0.0854	30.16
	(62.09)	(0.168)	(0.124)	(0.461)	(22.42)
*J Env Econ Mgt*	−4.130	0.377 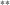	$-0.217^{*}$	$-1.705^{***}$	82.05 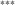
	(70.75)	(0.182)	(0.121)	(0.460)	(20.76)
*Climatic Change*	227.3 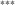	0.107	−0.0527	−0.358	75.84 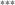
	(57.50)	(0.156)	(0.113)	(0.406)	(19.93)
*Env Res Econ*	−38.26	0.242	0.143	−0.140	45.71 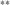
	(57.89)	(0.157)	(0.114)	(0.423)	(20.16)
Constant	−4,387	48.96 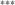	−0.949	92.96 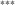	13,422 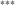
	(3420)	(8.988)	(6.360)	(22.53)	(1073)
Observations	787	807	871	911	1,078
*R*‐squared	0.139	0.086	0.047	0.057	0.175

*Note*: Standard errors are shown in parentheses; 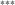 *p* <0.01; 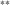 *p* <0.05; 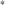 *p* <0.1.

Column 1 regresses the social cost of carbon on dummies for the 16 journals that published five papers or more. Columns 2–5 regress key assumptions on said dummies. All results are quality‐weighted and censored.

Table 3 repeats the exercise for the country of affiliation. There are no significant differences between countries. This is reassuring at the surface: Researchers from different places reach the same conclusion. However, it may be that researchers from unrepresented countries mimic the assumptions of researchers in the countries that dominate this literature (see the next section).

**TABLE 3:** Differences between countries.

	SCC	PRTP	EIS	Impact	Cited SCC
PRTP	$-115.7^{***}$				
	(15.74)				
EIS	−28.58				
	(19.82)				
Year	2.030	$-0.0261^{***}$	1.79e−05	$-0.0221^{*}$	$-7.551^{***}$
	(1.901)	(0.00517)	(0.00374)	(0.0113)	(0.645)
Austria	332.5	−0.197	0.236	−2.361	26.95
	(230.8)	(0.647)	(0.499)	(1.461)	(90.91)
China	179.0	0.482	−0.451	−0.443	84.63
	(208.8)	(0.577)	(0.446)	(1.416)	(76.68)
Finland	354.9	1.526 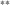	−1.79e−05	1.196	−0.466
	(263.0)	(0.735)	(0.567)	(1.686)	(104.9)
France	252.6	−0.338	−0.0695	−2.065	−13.19
	(214.6)	(0.580)	(0.451)	(1.299)	(78.41)
Germany	254.3	−0.333	−0.308	1.320	16.12
	(190.8)	(0.535)	(0.412)	(1.167)	(72.05)
Ireland	−44.83	−0.381	−0.257	3.108 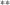	−59.41
	(204.2)	(0.572)	(0.441)	(1.275)	(78.53)
Italy	57.41	0.0542	1.188 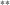	1.098	76.05
	(226.4)	(0.632)	(0.487)	(1.421)	(88.41)
Japan	−22.75	−0.0809	0.530	0.943	29.66
	(261.8)	(0.734)	(0.566)	(1.684)	(98.63)
Netherlands	312.3	0.657	−0.413	−1.135	100.3
	(260.8)	(0.731)	(0.494)	(1.410)	(83.23)
Norway	142.8	−0.0494	−0.198	0.821	147.9 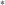
	(215.6)	(0.605)	(0.466)	(1.350)	(83.99)
Poland	143.3	0.157	−0.0308	1.126	352.7 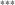
	(263.4)	(0.739)	(0.570)	(1.696)	(105.5)
South Korea					81.76
					(107.7)
Sweden	334.8	−0.838	−0.298	0.678	152.1 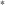
	(206.0)	(0.576)	(0.444)	(1.276)	(79.33)
Switzerland	−24.60	0.0523	−0.450		82.12
	(281.3)	(0.789)	(0.608)		(113.4)
UK	151.2	−0.145	−0.461	1.034	25.86
	(188.5)	(0.528)	(0.406)	(1.143)	(70.85)
USA	117.6	0.0534	−0.0447	0.873	33.63
	(185.9)	(0.521)	(0.402)	(1.129)	(70.03)
Constant	−3,805	54.23 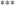	1.414	41.81 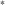	15,339 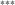
	(3845)	(10.45)	(7.545)	(22.79)	(1304)
Observations	556	571	615	637	775
*R*‐squared	0.154	0.102	0.087	0.095	0.191

*Note*: Standard errors are shown in parentheses; 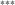 *p* <0.01; 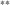 *p* <0.05; 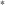 *p* <0.1.

Column 1 regresses the social cost of carbon on dummies for the countries of affiliation. The base category is multicountry. Columns 2–5 regress key assumptions on said dummies. All results are quality‐weighted and censored.

### Publication bias

Figure 4 and Table 2 show systematic differences between journals in the published estimates of the social cost of carbon. Table 2 tests for differences in the *assumptions* underlying these estimates. This would indicate that certain assumptions are frowned upon by a journal's referees or editors; or that authors believe that certain assumptions would increase their chance of getting published in that journal.

Three journals publish papers that assume a pure rate of time preference that is significantly higher and one journal lower than the rest. Three other journals have a more curved utility function, and one journal a flatter one. Two journals are more pessimistic about the impact of climate change. However, the two outlier journals identified in Figure 4 do not use statistically distinct assumptions. Their exceptional estimates are due to a combination of assumptions rather than a single one. There does not seem to be a consistent selection of papers on assumptions. The *Journal of Environmental Economics and Management*, for instance, publishes papers that are more pessimistic on climate change and use a lower inverse of the elasticity of intertemporal substitution (pushing the social cost of carbon up) but use a higher pure rate of time preference (pushing the social cost of carbon down).

Table 3 shows no statistically significant differences between countries, with the possible exception of authors based in Finland and Italy who are more impatient (second column) and risk‐averse (third column), respectively, than others; note that the sample size is small for these countries. Authors based in Ireland are especially optimistic about the impacts of climate change (fourth column). However, these differences do not lead to differences in the social cost of carbon (first column).

In sum, there is little evidence of *differential* publication bias. While it may be that certain results are more difficult to publish, it is hard to imagine that gatekeeping is consistent across countries and journals. The lack of evidence for differential gatekeeping militates against gatekeeping on results or assumptions.

### Citation bias

Table 1, column 5, shows that estimates of the social cost of carbon are not affected by the social cost of carbon in cited studies. Estimates in studies that are not cited but were cited in cited papers (“aware”) are significant at the 10% level, as are other noncited estimates. This suggests that authors preferentially cite lower estimates, perhaps to make their own estimates look more innovative. Dietz et al.^44^, for instance, announce a substantial revision of the social cost of carbon—their base estimate lies on the 35th percentile of previously published estimates, and their revised social cost of carbon lies on the 31st percentile.^45^ Alternatively, researchers may have tried to exclude authors of similar estimates from the pool of referees. The time trend becomes significant in this specification, as the noncited social cost of carbon falls over time.

Table 2, column 5, shows that five journals preferentially refer to papers with higher estimates of the social cost of carbon. One of these journals, *Climatic Change*, preferentially publishes papers with high estimates (column 1); the citation bias may reflect a preference for self‐citation. That explanation does not hold for the other four journals. No journal preferentially cites papers with lower estimates of the social cost of carbon.

Table 3 repeats this exercise for country of affiliation. Authors from Poland selectively cite pessimistic papers. The dummy for Sweden is significant at the 10% level. As with the journals, there are no significantly negative coefficients. While citation bias may not be widespread, it works in one direction only.

Table 4 returns to the right‐most column of Table 1. I regress the weighted average of the social cost of carbon in a paper on the average in the cited papers, the average in the papers that were cited in cited papers (“aware”) and in previously published papers that were not cited, controlling for the year of publication. In Table 1, the effect is significant at the 10% level. In Table 4, the effect of the social cost of carbon reported in the literature on the social cost of carbon is significant at the 5% level. As above, high estimates of the social cost of carbon are associated with high estimates in noncited papers. The effect is large: if the social cost of carbon is $10/tC higher in noncited (but known) papers, the reported social cost of carbon increases by $10/tC ($5/tC). This suggests that people preferentially cite lower estimates to make their own estimate look novel.

**TABLE 4:** Evidence of citation bias.

	SCC	PRTP	EIS	Benchmark	Citations
Cited	−0.0223	0.00877	0.0956	0.0440	
	(0.115)	(0.118)	(0.106)	(0.117)	
Aware	0.462 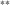	−0.267	$-0.241^{*}$	0.0161	
	(0.205)	(0.232)	(0.129)	(0.289)	
Not	0.956 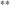	0.931	1.146 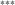	1.636 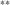	
	(0.461)	(0.577)	(0.172)	(0.717)	
Year	5.457 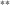	−0.0102	0.0125 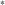	−0.0117	$-0.0544^{***}$
	(2.191)	(0.0134)	(0.00697)	(0.0131)	(0.0125)
SCC					−0.000766
					(0.000511)
PRTP					−0.162
					(0.133)
EIS					−0.282
					(0.208)
Benchmark					$-0.105^{**}$
					(0.0508)
lnα					0.520 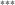
					(0.117)
Constant	$-11,052^{**}$	20.88	$-25.21^{*}$	24.33	112.1 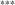
	(4456)	(27.81)	(14.22)	(26.10)	(25.27)
Observations	317	190	230	247	178
*R*‐squared	0.034	0.094	0.244	0.034	

*Note*: Standard errors are shown in parentheses; 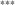 *p* <0.01; 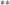 *p* <0.05; 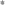 *p* <0.1.

Columns 1–4 regress the social cost of carbon (SCC), pure rate of time preference (PRTP), inverse of the elasticity of intertemporal substitution (EIS), and impact of 2.5℃ warming, average over all estimates in a paper, on the same indicators averaged over all papers cited, over papers not cited but cited in the cited papers (“aware”), and over other papers not cited. Column 5 uses a negative binomial regression of the number of citations on the characteristics of the cited paper; the estimate for lnα indicates that this specification outperforms a Poisson regression.

This is a paradox. Tables 1 and 4 show that high estimates are not cited, while Table 2 shows that citing high estimates increases the chance of publication. However, as the latter result only holds for some journals, the two results can be reconciled by authors avoiding citing high estimates unless they target the journals identified in Table 2.

The assumed pure rate of time preference does not depend on assumptions in previous studies. However, the elasticity of intertemporal substitution and the impact of 2.5℃ warming do. Particularly, authors who use a high inverse of the elasticity of intertemperal substitution appear to avoid citing studies that do the same. Similarly, studies that have a more optimistic outlook on the impact of 2.5℃ global warming cite similar studies less.

The rightmost column of Table 4 confirms the last result. It regresses the number of citations on study characteristics. Papers that are less pessimistic about the impact of climate change receive fewer citations. These studies report lower estimates of the social cost of carbon (Table 1) but citation selection is on the total impact of climate change rather than the marginal impact.

## CONCLUSION

I update and extend the meta‐analysis of the social cost of climate change.^5^ I confirm that the uncertainty about the social cost of carbon is large and right‐skewed. The overall mean estimate is $207/tC ($759/tCO_2_). Estimates have trended upward over time, primarily because of a shift in the ethical positions of the analysts. The social cost of carbon is higher if the impact of climate change is worse or if the discount rate is lower. The extension of the meta‐database reveals that the literature on the social cost of carbon is dominated by a few networks of co‐authors, who are predominantly based in Europe and North America. The social cost of carbon is a recommendation for a *global* carbon tax. It would be easier for leaders in other parts of the world to accept this if their researchers would contribute to the literature. Furthermore, preferences may differ across the world.^25^

There is some evidence of publication bias: Some journals prefer to publish higher estimates of the social cost of carbon. This raises the overall average. There is evidence of citation bias too. Authors systematically ignore earlier, higher estimates, perhaps because this allows them to present their own, higher estimates as novel.

The following caveats apply. Any meta‐analysis is only as good as the underlying database. New estimates of the social cost of carbon are published frequently. Methods to estimate the social cost of carbon change, so new fields must be added to the database. Here, I coded broad similarities rather than fine differences between methods and assumptions. Others may take a different approach. The meta‐analysis is limited to the mean. Quantile regression, semi‐ or nonparametric regression, or machine learning methods may lead to somewhat different conclusions.

The results presented here are associations. Causality is hard to establish without multiple iterations of each paper^46^, ^47^ and referee reports.^48^ Controlled experiments are impractical and unethical—but see Huber et al.^49^ In this case, natural experiments were uninformative.

These things can be deferred to future research. For now, three key findings emerge: (1) The social cost of carbon is large and rising. This justifies greenhouse gas emission reduction. (2) The literature on the social cost of carbon suffers from publication or citation bias, exaggerating estimates. (3) The limited geographical spread of researchers may affect the political acceptability of these findings.

## CONFLICT OF INTEREST STATEMENT

The author declares no conflict of interests.

## PEER REVIEW

The peer review history for this article is available at https://publons.com/publon/10.1111/nyas.15340.

## Supporting information



Supporting Information

## Footnotes

a The database has an indicator for uncertainty,^15^, ^16^ but not for stochasticity.^17^, ^18^ Stochastic models are calibrated to overestimate the social cost of carbon.^19^

b The social cost of carbon is the monetized value of the first partial derivative of the intertemporal welfare function to carbon dioxide emissions in a particular year. This derivative can be solved analytically, it equals the carbon tax in an optimization problem, it is an element of the Jacobean of an optimization problem, or it can be approximated as a small increment in a simulation model. Some papers report an average rather than a marginal, or report a social cost of carbon that does not appear to be based on any analytical or numerical model.

c Nordhaus^32^ too used an analytic approximation.

d Note that, from Nordhaus^40^ onward, climate change has been assumed to affect economic growth; econometric studies find much larger effects than calibrated models,^41^ perhaps because the econometric models are misspecified.^42^, ^43^

## References

[1] Greenstone, M. , Kopits, E. , & Wolverton, A. (2013). Developing a social cost of carbon for US regulatory analysis: A methodology and interpretation. Review of Environmental Economics and Policy, 7(1), 23–46.

[2] Pizer, W. A. , Adler, M. , Aldy, J. , Anthoff, D. , Cropper, M. , Gillingham, K. , Greenstone, M. , Murray, B. , Newell, R. , Richels, R. , Rowell, A. , Waldhoff, S. , & Wiener, J. (2014). Using and improving the social cost of carbon. Science, 346(6214), 1189–1190. 25477446 10.1126/science.1259774

[3] NAS . (2017). Valuing climate damages: Updating estimation of the social cost of carbon dioxide. Washington, DC: National Academies of Sciences, Engineering, and Medicine.

[4] Pezzey, J. C. V. (2019). Why the social cost of carbon will always be disputed. WIREs Climate Change, 10(1), e558.

[5] Tol, R. S. J. (2023). Social cost of carbon estimates have increased over time. Nature Climate Change, 13, 532–536.

[6] Tol, R. S. J. (2005). The marginal damage costs of carbon dioxide emissions: An assessment of the uncertainties. Energy Policy, 33, 2064–2074.

[7] Tol, R. S. J. (2011). The social cost of carbon. Annual Review of Resource Economics, 3, 419–443.

[8] Wang, P. , Deng, X. , Zhou, H. , & Yu, S. (2019). Estimates of the social cost of carbon: A review based on meta‐analysis. Journal of Cleaner Production, 209, 1494–1507.

[9] Havránek, T. , Irsova, Z. , Janda, K. , & Zilberman, D. (2015). Selective reporting and the social cost of carbon. Energy Economics, 51, 394–406.

[10] Tol, R. S. J. (2018). The economic impacts of climate change. Review of Environmental Economics and Policy, 12(1), 4–25. 10.1093/reep/rez021 PMC7147821 32280366

[11] Anthoff, D. , & Tol, R. S. J. (2022). Testing the dismal theorem. Journal of the Association of Environmental and Resource Economists, 9(5), 885–920.

[12] Nieminen, P. , Rucker, G. , Miettunen, J. , Carpenter, J. , & Schumacher, M. (2007). Statistically significant papers in psychiatry were cited more often than others. Journal of Clinical Epidemiology, 60(9), 939–946. 17689810 10.1016/j.jclinepi.2006.11.014

[13] Radicchi, F. , & Castellano, C. (2012). A reverse engineering approach to the suppression of citation biases reveals universal properties of citation distributions. PLoS ONE, 7(3), e33833. 22479454 10.1371/journal.pone.0033833 PMC3315498

[14] Jannot, A.‐S. , Agoritsas, T. , Gayet‐Ageron, A. , & Perneger, T. V. (2013). Citation bias favoring statistically significant studies was present in medical research. Journal of Clinical Epidemiology, 66(3), 296–301. 23347853 10.1016/j.jclinepi.2012.09.015

[15] Plambeck, E. L , & Hope, C. W. (1996). Page95: An updated valuation of the impacts of global warming. Energy Policy, 24(9), 783–793.

[16] Tol, R. S. J. (1999). The marginal costs of greenhouse gas emissions. Energy Journal, 20(1), 61–81.

[17] Cai, Y. , & Lontzek, T. S. (2019). The social cost of carbon with economic and climate risks. Journal of Political Economy, 127(6), 2684–2734.

[18] van den Bremer, T. S , & van der Ploeg, F. (2021). The risk‐adjusted carbon price. American Economic Review, 111(9), 2782–2810.

[19] Estrada, F. , & Tol, R. S. J. (2015). Toward impact functions for stochastic climate change. Climate Change Economics, 6(04), 1–13.

[20] Tol, R. S. J. (2012). Leviathan taxes in the short run. Climatic Change Letters, 113(3–4), 1049–1063.

[21] Tol, R. S. (2022). A meta‐analysis of the total economic impact of climate change. Working Paper Series 0422, Department of Economics, University of Sussex Business School.

[22] Rennert, K. , Errickson, F. , Prest, B. C. , Rennels, L. , Newell, R. G. , Pizer, W. A. , Kingdon, C. , Wingenroth, J. , Cooke, R. , Parthum, B. , Smith, D. , Cromar, K. , Diaz, D. B. , Moore, F. C. , Müller, U. K. , Plevin, R. J. , Raftery, A. E. , S̆evc̆íková, H. , Sheets, H. , … Anthoff, D. (2022). Comprehensive evidence implies a higher social cost of CO_2_ . Nature, 610, 687–692. 36049503 10.1038/s41586-022-05224-9 PMC9605864

[23] Nordhaus, W. D. (1980). Thinking about carbon dioxide: Theoretical and empirical aspects of optimal control strategies. Discussion Paper 565, Cowles Foundation for Research in Economics.

[24] Barrage, L. , & Nordhaus, W. D. (2023). Policies, projections, and the social cost of carbon: Results from the DICE‐2023 model. Cowles Foundation Discussion Papers 2363, Cowles Foundation for Research in Economics, Yale University. 10.1073/pnas.2312030121 PMC10990119 38502689

[25] Dong, J. , Tol, R. S. , & Wang, F. (2024). Towards a social cost of carbon with national characteristics. Economics Letters, 244, 111977.

[26] Nordhaus, W. D. (1991). To slow or not to slow: The economics of the greenhouse effect. Economic Journal, 101(444), 920–937.

[27] Nordhaus, W. D. (1994). Managing the global commons: The economics of climate change. Cambridge: MIT Press.

[28] Stern, N. H. , Peters, S. , Bakhski, V. , Bowen, A. , Cameron, C. , Catovsky, S. , Crane, D. , Cruickshank, S. , Dietz, S. , Edmondson, N. , Garbett, S.‐L. , Hamid, L. , Hoffman, G. , Ingram, D. , Jones, B. , Patmore, N. , Radcliffe, H. , Sathiyarajah, R. , Stock, M. , … Zenghelis, D. (2006). Stern review: The economics of climate change. London: HM Treasury.

[29] Nordhaus, W. D. (2008). A question of balance—Weighing the options on global warming policies. New Haven, CT: Yale University Press.

[30] Peck, S. C. , & Teisberg, T. J. (1992). CETA: A Model for Carbon Emissions Trajectory Assessment. Energy Journal, 13, 55–78.

[31] Golosov, M. , Hassler, J. , Krusell, P. , & Tsyvinski, A. (2014). Optimal taxes on fossil fuel in general equilibrium. Econometrica, 82(1), 41–88.

[32] Nordhaus, W. D. (1982). How fast should we graze the global commons? American Economic Review, 72(2), 242–246.

[33] van den Bijgaart, I. , Gerlagh, R. , & Liski, M. (2016). A simple formula for the social cost of carbon. Journal of Environmental Economics and Management, 77, 75–94.

[34] Ramsey, F. P. (1928). A mathematical theory of saving. Economic Journal, 38(152), 543–559.

[35] Anthoff, D. , Tol, R. S. J. , & Yohe, G. W. (2009). Risk aversion, time preference, and the social cost of carbon. Environmental Research Letters, 4(2), 024002.

[36] Dell, M. , Jones, B. F. , & Olken, B. A. (2012). Temperature shocks and economic growth: Evidence from the last half century. American Economic Journal: Macroeconomics, 4(3), 66–95.

[37] Burke, M. , Hsiang, S. M. , & Miguel, E. (2015). Global non‐linear effect of temperature on economic production. Nature, 527(7577), 235–239. 26503051 10.1038/nature15725

[38] Moyer, E. J. , Woolley, M. D. , Matteson, N. J. , Glotter, M. J. , & Weisbach, D. A. (2014). Climate impacts on economic growth as drivers of uncertainty in the social cost of carbon. Journal of Legal Studies, 43(2), 401–425.

[39] Moore, F. C , & Diaz, D. B. (2015). Temperature impacts on economic growth warrant stringent mitigation policy. Nature Climate Change, 5(2), 127–131.

[40] Nordhaus, W. D. (1992). An optimal transition path for controlling greenhouse gases. Science, 258, 1315–1319. 17778354 10.1126/science.258.5086.1315

[41] Fankhauser, S. , & Tol, R. S. J. (2005). On climate change and economic growth. Resource and Energy Economics, 27(1), 1–17.

[42] Newell, R. G. , Prest, B. C. , & Sexton, S. E. (2021). The GDP‐temperature relationship: Implications for climate change damages. Journal of Environmental Economics and Management, 108, 102445.

[43] Tol, R. S. J. (2024). A meta‐analysis of the total economic impact of climate change. Energy Policy, 185, 113922.

[44] Dietz, S. , van der Ploeg, F. , Rezai, A. , & Venmans, F. (2021). Are economists getting climate dynamics right and does it matter? Journal of the Association of Environmental and Resource Economists, 8(5), 895–921.

[45] Tol, R. S. J. (2021). Do climate dynamics matter for economics? Nature Climate Change, 11(10), 802–803.

[46] Hengel, E. (2022). Publishing while female: Are women held to higher standards? Evidence from peer review. Economic Journal, 132(648), 2951–2991.

[47] Aranzales Acero, I. D. (2024). Knowledge creation, dissemination and disappearance: Exploring the interplay of emotions, creativity and academic publishing in knowledge production. Brisbane: Queensland University of Technology.

[48] Alexander, D. , Gorelkina, O. , Hengel, E. , & Tol, R. S. J. (2023). Gender and the time cost of peer review. Tinbergen Institute Discussion Papers 23‐044/V, Tinbergen Institute.

[49] Huber, J. , Inoua, S. , Kerschbamer, R. , König‐Kersting, C. , Palan, S. , & Smith, V. L. (2022). Nobel and novice: Author prominence affects peer review. Proceedings of the National Academy of Sciences, 119(41), e2205779119. 10.1073/pnas.2205779119 PMC9564227 36194633

