# Supplementary material for: Trends and biases in the social cost of carbon
Source: Ann N Y Acad Sci. 2025 May 7;1548(1):248–59. doi: 10.1111/nyas.15340 (PMC12220299; doi:10.1111/nyas.15340)
Supplement: Supplementary file 1 — Supporting Information [file NYAS-1548-248-s001.pdf]

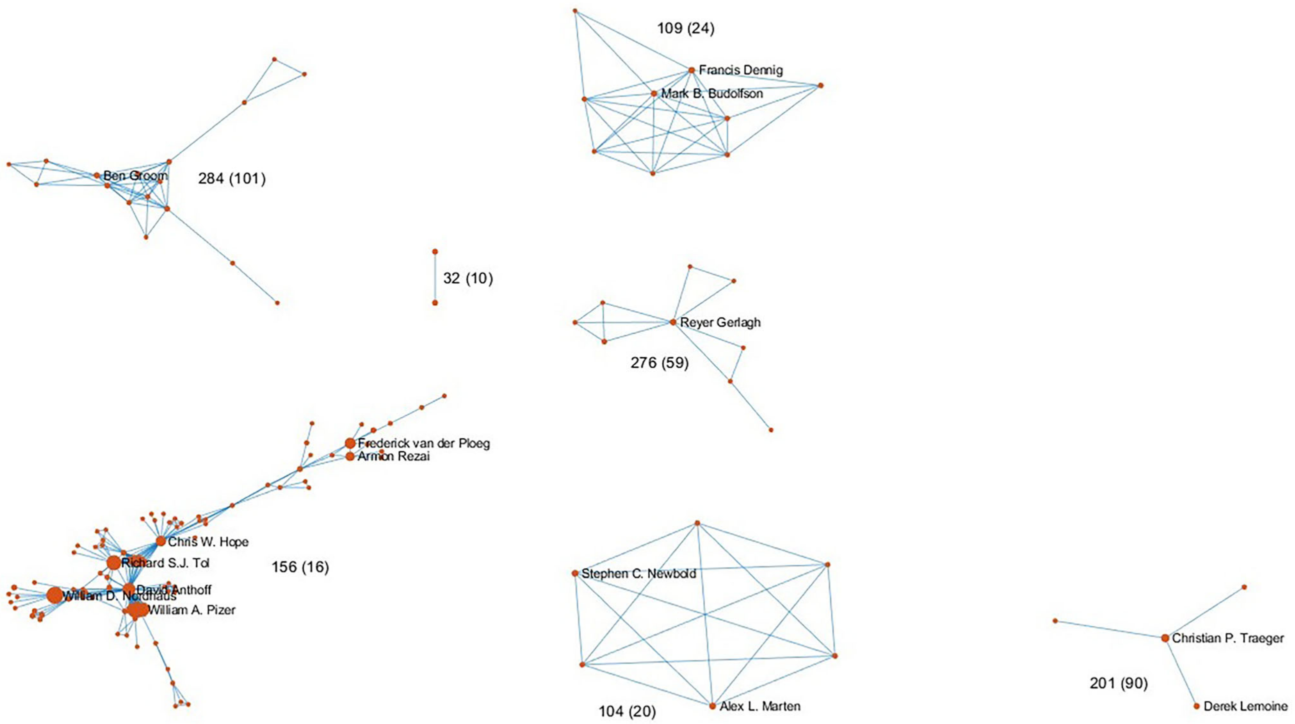

FIGURE S1 The seven most prolific co-author networks. Node size is the number of co-authored papers. Authors of six papers or more are named. The numbers are the average social cost of carbon and its standard error for papers published by authors in the network.

ADDITIONAL RESULTS

Figure S2 is hard to read. Figure S3 reveals why. It is a subgraph limited to papers by Nordhaus. This much smaller graph is hard to read too. Because of the complexity in citation patterns, the network in Figure S2 resists clustering. Table S1 shows correlations between citations to eight groups of papers (counting Golosov et al.[31] as a group of one). There are some patterns: Papers by Chris Hope and the US Interagency Working Group on the Social Cost of Carbon are often cited together. That seems to make sense as the Working Group relied on Hope's model—but they also relied on the models of Nordhaus and Tol, whose papers show a mild *anti*correlation. Stern also relied on Hope, but papers citing Stern do not acknowledge that. In sum, the citation network of 323 papers is complex. There are no discernible groups of papers that would simplify the visualization.

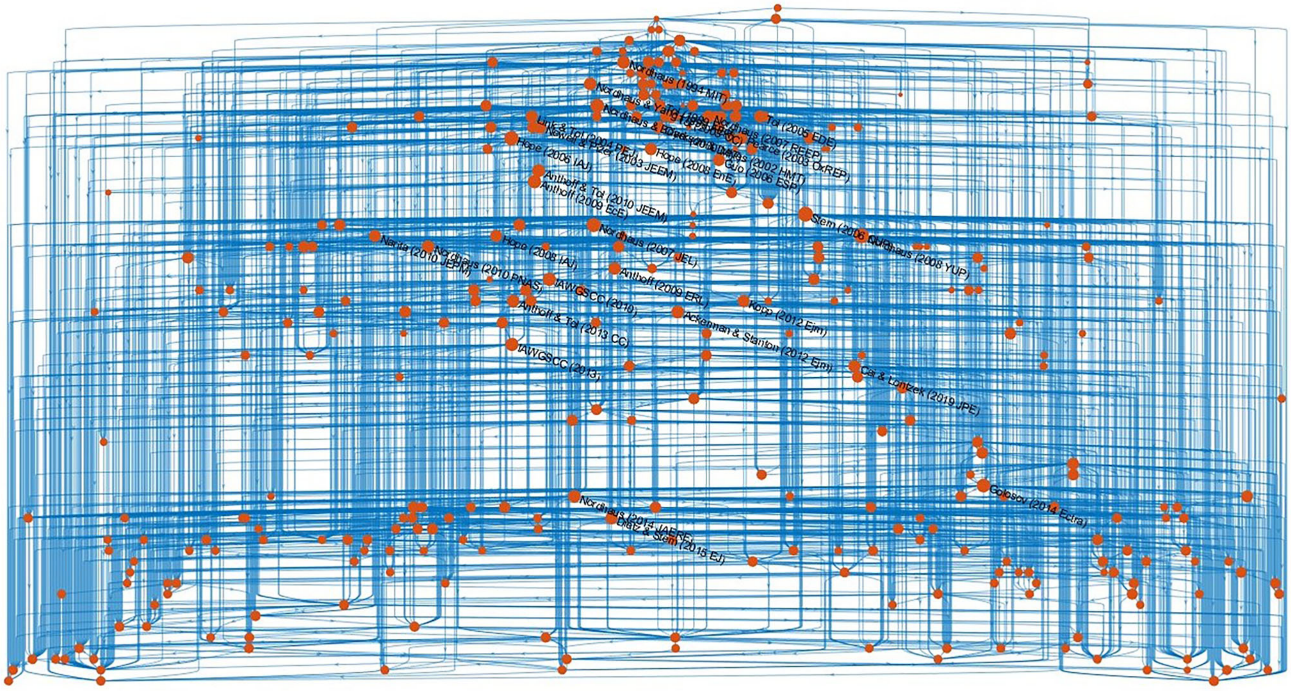

FIGURE S2 The citation network of papers on the social cost of carbon. Node size is incloseness corrected for number of citation and age.

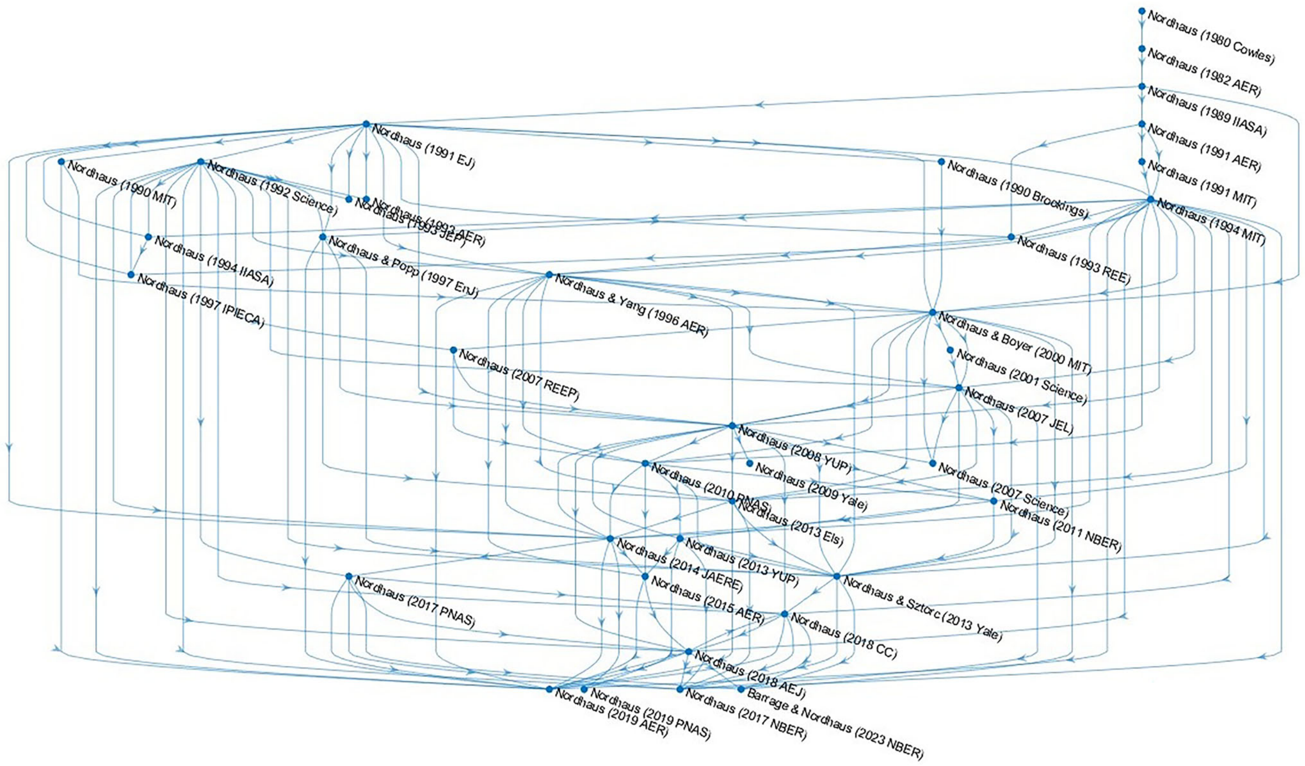

FIGURE S3 Nordhaus citing Nordhaus. The citation network of papers by William D. Nordhaus on the social cost of carbon.

TABLE S1 Correlations between citations.

		Nordhaus	Hope	Tol	Stern	US Gov't	Ploeg	Traeger	Golosov
1980	Nordhaus	1							
1996	Hope	–0.06	1						
1999	Tol	0.20	–0.03	1					
2006	Stern	0.23	0.06	0.14	1				
2009	US Gov't	–0.16	0.53	–0.21	0.05	1			
2013	Ploeg	–0.07	0.08	–0.01	0.16	0.09	1		
2013	Traeger	0.29	–0.07	0.52	0.13	–0.17	–0.11	1	
2014	Golosov	–0.11	0.34	–0.14	0.14	0.44	0.09	–0.12	1
